# The Effect of Prucalopride on Small Bowel Transit Time in Hospitalized Patients Undergoing Capsule Endoscopy

**DOI:** 10.1155/2017/2696947

**Published:** 2017-11-23

**Authors:** Majid Alsahafi, Paula Cramer, Nazira Chatur, Fergal Donnellan

**Affiliations:** ^1^Division of Gastroenterology, Vancouver General Hospital, University of British Columbia, Vancouver, BC, Canada; ^2^Department of Medicine, King Abdulaziz University, Jeddah, Saudi Arabia

## Abstract

**Background:**

The inpatient status is a well-known risk factor for incomplete video capsule endoscopy (VCE) examinations due to prolonged transit time. We aimed to evaluate the effect of prucalopride on small bowel transit time for hospitalized patients undergoing VCE.

**Methods:**

We included all hospitalized patients who underwent VCE at a tertiary academic center from October 2011 through September 2016. A single 2 mg dose of prucalopride was given exclusively for all patients who underwent VCE between March 2014 and December 2015. VCE studies were excluded if the capsule was retained or endoscopically placed, if other prokinetic agents were given, in cases with technical failure, or if patients had prior gastric or small bowel resection.

**Results:**

442 VCE were identified, of which 68 were performed in hospitalized patients. 54 inpatients were included, of which 29 consecutive patients received prucalopride. The prucalopride group had a significantly shorter small bowel transit time compared to the control group (92 versus 275.5, *p* < 0.001). There was a trend for a higher completion rate in the prucalopride group (93.1% versus 76%, *p* = 0.12).

**Conclusions:**

Our results suggest that the administration of prucalopride prior to VCE is a simple and effective intervention to decrease small bowel transit time.

## 1. Introduction

Small bowel video capsule endoscopy (VCE) has revolutionized the management of small bowel disorders and is considered now the first line to investigate for obscure gastrointestinal bleeding [1–5]. Optimal diagnostic utility depends not only on adequate small bowel visualization, but also on the completion of small bowel examination. Because of the limited battery time, 16.5% of VCE studies are incomplete [6]. Prolongation of the small bowel transit may result in lower completion rates, which limits the diagnostic utility of VCE and increases the cost associated with further diagnostic testing.

Inpatient VCE is commonly performed to investigate the source of obscure gastrointestinal bleeding. The inpatient status is a well-known risk factor for incomplete small bowel examination with a reported completion rate as low as 64%, limiting the diagnostic utility of VCE [7–11]. The performance of VCE after hospital discharge is potentially associated with a decrease in the diagnostic yield, delays therapeutic interventions, and is not always possible. Several studies have shown that VCE performed during or early after the bleeding event is associated with a higher diagnostic yield [10, 12–15]. Therefore, interventions to shorten small bowel transit time are particularly needed for hospitalized patients to increase the completion rates. The experience with purgatives and prokinetic agents revealed mixed results with no conclusive benefit [16–18].

Prucalopride is a 5-HT4 receptor agonist that has been shown to improve colonic motility. It has been approved in Canada and Europe for treatment of chronic idiopathic constipation in women who fail to respond to laxatives. In addition to improving colonic motility, animal and human studies suggest that prucalopride stimulates motility in the stomach and small intestine, and as a result it has the potential to decrease the transit times in the stomach and the small bowel [19–24]. The effect of prucalopride on small bowel transit time (SBTT) in patients undergoing VCE has not been previously investigated. We therefore aimed to determine the effect of prucalopride on SBTT for hospitalized patients who are particularly at risk for prolongation of SBTT.

## 2. Methods

### 2.1. Patients

The study was approved by the University of British Columbia Clinical Research Ethics Board and the Vancouver Coastal Health Research Institute. We included all hospitalized patients who underwent VCE at Vancouver General Hospital from October 2011 through September 2016. Vancouver General Hospital is a tertiary, academic center and the largest hospital in British Columbia, Canada. Inpatient VCE were identified through a prospective capsule endoscopy database that includes data related to all patients who underwent VCE at our center. The database includes information related to patients' demographics, indication for VCE, use of prokinetics, and completeness of small bowel examination. VCE studies were excluded if the capsule was retained or endoscopically placed, if other prokinetic agents were given, or if the patient had prior gastric or small bowel resection. Capsule retention is defined as the presence of the capsule endoscope in the digestive tract for a minimum of 2 weeks or more, or when the capsule is retained indefinitely in the small bowel unless a targeted medical or surgical intervention is initiated. VCE studies with technical failure, including accidental removal of the sensor/recorder, were also excluded. In addition to the data recorded in the database, we retrospectively reviewed the hospital electronic medical records, pre-VCE clinical assessment forms, and the VCE reports to collect data on medical comorbidities and the use of the following medications: narcotics, antiplatelet drugs, nonsteroidal anti-inflammatory drugs, warfarin, and other anticoagulants.

### 2.2. VCE Procedure

Prior to January 2015, all small bowel VCE studies were performed using Endocapsule EC1 (Olympus, Tokyo, Japan). After this date, all VCE studies were performed using Pillcam SB3 (Given Imaging, Yokneam, Israel). Patients were given standard instructions as per our usual practice including a clear liquid diet after lunch the day prior to VCE, followed by an overnight fast. They were permitted to resume a clear fluid diet and to have a light meal two and four hours after the beginning of recording, respectively. Patients received 2 L of polyethylene glycol starting at 4 pm the day before the procedure. Between March 2014 and December 2015, all hospitalized patients were given a 2 mg single dose of prucalopride orally at the time of VCE as per our protocol during this period. No patient received prucalopride outside this period. Apart from the bowel preparation, no other mucosal cleansing or antibubbles agents were given. All patients had the VCE recorders disconnected after 8 hours of recording.

### 2.3. VCE Interpretation

Data related to VCE interpretation was collected from the VCE reports. One of two gastroenterologists with experience in VCE reviewed and interpreted the VCE images at a maximum rate of 15 images per second as per our usual practice. The reviewers were not blinded to clinical data but they were unaware of the study hypothesis as the study hypothesis was conceived after all VCE were done. We collected data on gastric transit time (GTT), SBTT, small bowel completion rate, and the diagnostic yield. SBTT was defined as the time from the first duodenal image to the first cecal image. GTT was defined as the time from the first gastric image to the first duodenal image.

Small bowel completion rate and the diagnostic yield were defined as the proportion of VCE in which the cecum was reached and the proportion of VCE with positive findings, respectively. The findings on VCE were categorized as positive or negative using a modified version of a previously reported P0–P2 system [25]. The VCE study was considered positive if a significant lesion (P2) was reported. The presence of fresh blood of unclear source was considered a positive finding as this suggests the site of bleeding and helps with further management. Alternatively, the VCE study was considered negative if no abnormality was found (P0) or an abnormality of uncertain significance was reported (P1). Abnormalities of uncertain significance included minor isolated erosions and small nonspecific red spots.

### 2.4. Statistical Analysis

The mean and standard deviation and/or the median with range were used for continuous variables as appropriate. The percentage and count were used for categorical variables. Statistical analysis was performed with the Chi-square test or Fisher exact test for categorical variables as appropriate. The SBTT and GTT were compared between the two groups using the Wilcoxon Rank Sum test. A *p* value of less than 0.05, with a two-tail test, was considered statistically significant. Statistical analysis was performed using the R statistical software, version 0.99.893, © 2009–2016 R Studio, Inc.

## 3. Results

A total of 442 VCE were identified in our capsule endoscopy database, of which 68 were performed in hospitalized patients. Twelve VCE were excluded for the following reasons: 6 patients had endoscopic placements including 2 with previous gastric surgery; 2 had capsule retention; 2 had VCE with technical failure; 1 received linaclotide; 1 patient had prior small bowel resection. In addition, two patients had insufficient data and were excluded. Therefore, 54 VCE were included in the analysis, in which 29 consecutive patients received prucalopride. Twenty-six VCE were performed using Endocapsule (17 in the control and 9 in the prucalopride) and 28 using Pillcam (8 in the control and 20 in the prucalopride). For the entire study population, the mean age was 64.8 years (±15) and 64.8% were male.

The baseline characteristics for patients who received prucalopride versus those who did not receive prucalopride are shown in Table 1. There were no differences between the prucalopride and the control group concerning age, gender, indication for VCE, medical comorbidities, antiplatelets, anticoagulants, nonsteroidal anti-inflammatory drugs, or narcotics use. All patients tolerated the procedure well with no adverse events.

**Figure 1:**
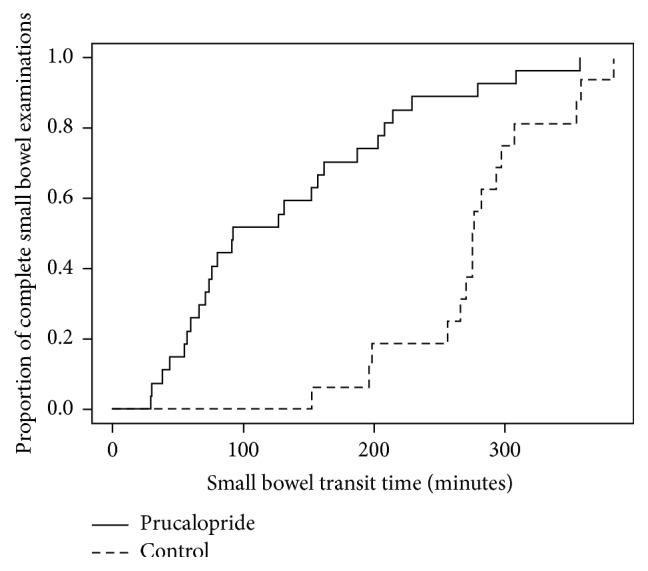
Kaplan-Meier curves for time to complete small bowel examination since the first duodenal image for all VCE that reached cecum. The median SBTT was significantly shorter in the prucalopride group (92, 95% CI: 74, 187) compared to the control group (275.5, 95% CI: 266, 354).* VCE, video capsule endoscopy; SBTT, small bowel transit time; CI, confidence* interval.

**Figure 2:**
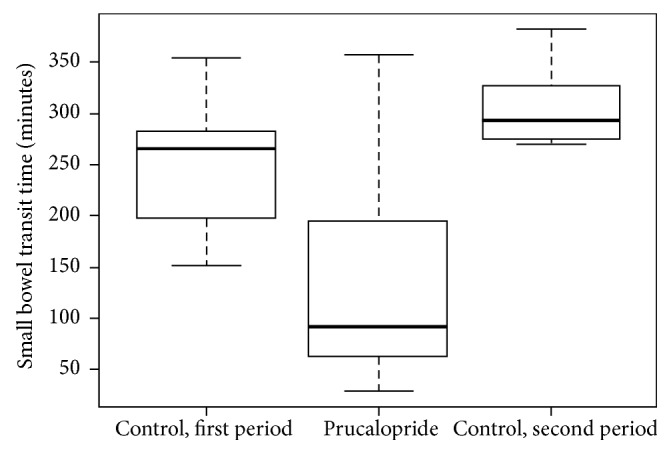
Box plot diagrams showing the distributions of small bowel transit time in the three study periods. Control, first period (October 2011 to March 2014); the period of prucalopride use (from March 2014 to December 2015); control, second period (December 2015 to September 2016).

The results of the study are summarized in Table 2. In the entire study population, the median SBTT was 196 minutes (IQR 78–275.5, range 29–383). The median SBTT was significantly shorter in the prucalopride group (92 versus 275.5 minutes, *p* < 0.001). To exclude any effect related to an unapparent change in our practice during the study period, we reanalyzed the data after excluding all VCE that were performed prior January 2015 (26 VCE). In the remaining 28 VCE included in this analysis, all VCE were performed using the Pillcam VCE. This analysis also revealed a significantly shorter median SBTT in the prucalopride group (85 versus 293 minutes, *p* < 0.001).  Figure 1 shows the Kaplan-Meier curves for time to complete small bowel examination for the prucalopride versus the control group.  Figure 2 showed the distributions of SBTT before the introduction of prucalopride, during the period of prucalopride use and after the end of prucalopride period.

**Figure 3:**
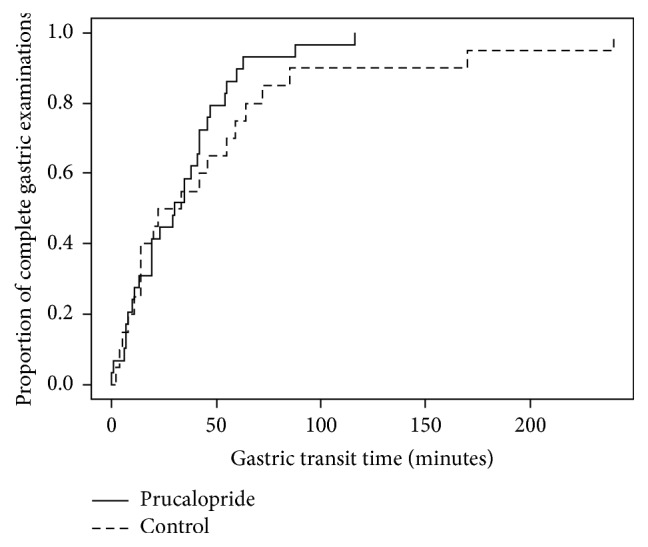
Kaplan-Meier curves for time to capsule passage into duodenum since the first gastric image for all VCE that reached duodenum. The median GTT was not significantly different between the prucalopride group (30, 95% CI: 19, 42) and the control group (27.5, 95% CI: 14, 72).* VCE, video capsule endoscopy; GTT, gastric transit time; CI, confidence* interval.

The capsule remained in the stomach only in 1 patient and this was in the control group. In the entire study population, the median GTT was 30 minutes (IQR 11–54, range 0–240). There was no difference in the median GTT between the prucalopride and the control group (30 versus 27.5, *p* = 0.61). Similarly, there was no difference in the GTT between VCE with complete and incomplete small bowel examination (median GTT was 30 minutes for both, *p* = 0.85).  Figure 3 shows the Kaplan-Meier curves for time to gastric passage for the prucalopride versus the control group.

Of the 54 VCE, the cecum was reached in 46 which resulted in an overall completion rate of 85.1%. The completion rate was higher in the prucalopride group (93.1%, 27/29) compared to the control group (76%, 19/25), *p* = 0.12. The overall diagnostic yield was 61.1% (33/54). Positive findings are summarized in Table 3. The diagnostic yield was higher in the prucalopride group (75.8%, 22/29) compared to the control group (44%, 11/25), *p* = 0.03. There was a trend for a higher diagnostic yield in complete VCE (65.2%, 30/46) compared to incomplete VCE (37.5%, 3/8), *p* = 0.23.

## 4. Discussion

Incomplete small bowel examination is an important limitation for the diagnostic utility of VCE. Several factors have been recognized as potential risk factors for incomplete VCE including the inpatients status, prior small bowel surgery, prolonged gastric passage, older age, diabetes, and poor bowel preparation [7–9, 26]. Hospitalized patients are particularly at risk for incomplete VCE, with a completion rate in the range of 64 to 73% [7–11]. One potential strategy is to accelerate the passage of the capsule through the stomach and small bowel preventing prolonged transit time. The use of purgatives does not appear to affect the transit times or the completion rates [17, 18]. Several prokinetic agents, such as erythromycin and metoclopramide, have been investigated but the evidence remains inconclusive [16, 17]. Magnetic manipulation of the VCE was not successful to shorten the GTT, while limited data suggests that the right lateral position might be of some benefit to increase the completion rate [27, 28]. The alternative strategy is to extend the battery life. However, even when the battery life is extended to 16 hours, 10% of VCE are incomplete [29].

The primary objective of our study was to examine if prucalopride, given at the time of VCE ingestion, decreases small bowel transit time in hospitalized patients. We found that prucalopride significantly decreased small bowel transit time. The median SBTT was 92 minutes in the prucalopride group and 275.5 minutes in the control group, *p* < 0.001. Patients in the prucalopride group were more likely to have complete small bowel examination, 93.1% versus 76%, although this did not reach the level of statistical significance (*p* = 0.12) as our study was not adequately powered to assess a difference in the completion rates.

Our study is the first study to evaluate the effect of prucalopride on SBTT in patients undergoing VCE. While the effect of prucalopride on the colonic motility is well described in humans, its effect on small bowel motility has been less studied. Animal studies suggest that prucalopride stimulates small intestinal motility [19, 20]. Studies in healthy volunteers were limited by small size and revealed mixed results. In a cross-over study that included healthy volunteers, prucalopride significantly decreased the transit time from mouth to cecum [22]. In contrast, in another small study that also included healthy subjects, prucalopride did not significantly affect gastric or small bowel transit time [23]. However, in a randomized controlled trial that included patients with constipation, who are likely to be at a higher risk incomplete VCE studies, prucalopride has been shown to decrease both the gastric and the small bowel transit times [24].

In the present study, the SBTT in the prucalopride group was substantially shorter than the SBTT reported in the literature for both inpatient and outpatient VCE. Furthermore, the completion rate in the prucalopride group was also substantially higher than the completion rate reported in the literature for the inpatient VCE and is comparable to the completion rates for outpatient VCE [9–11]. For the control group, the SBTT and completion rate were similar to what is reported in the literature [7–9, 11]. Therefore, the differences in the SBTT and the completion rates between the two groups in our study did not result from disproportionately longer SBTT and lower completion rate in the control group biasing the results in favor of the prucalopride group. The significant difference in the SBTT persisted after we excluded all VCE that were done prior to January 2015 indicating that the difference was not resulting from an unapparent change in our practice in the five-year period of the study. Furthermore, as shown in Figure 2, the marked decrease in the SBTT after the introduction of prucalopride was followed by a marked increase in the SBTT when prucalopride was no longer used, suggesting a causative effect of prucalopride rather than just an association. The bivariate analysis revealed no differences in the baseline characteristics between the two study groups.

Interestingly, the increase in the completion rate in the prucalopride group resulted from accelerating the small bowel transit rather than the gastric passage, as there was no difference in the GTT between the prucalopride group and the control group. Previous studies evaluating the effect of prucalopride on gastric emptying revealed controversial results [21, 23, 24]. While prucalopride did not significantly affect the GTT in our study, it is unclear if prucalopride would accelerate the passage of the capsule in a subset of patients who have prolonged GTT, such as patients with diabetic gastroparesis. It is worth noting that we administered prucalopride at the time of capsule ingestion, which may not have been the ideal time to influence the GTT as the maximum plasma concentration usually occurs after 2.7 hours of ingestion [30].

The ultimate goal of decreasing SBTT is to increase the rate of complete examinations, thus enhancing the diagnostic utility of VCE by increasing the diagnostic yield and/or eliminating the uncertainty related to incomplete examinations. Two published studies associated higher diagnostic yields with prolonged SBTT raising concerns about the routine use of prokinetic agents [31, 32]. While there are limitations for both studies, including the retrospective design and the absence of strict definitions for positive findings, theoretically there is more opportunity to miss lesions when fewer images are obtained as a result of rapid transit. However, when the prolonged transit is the cause for incomplete examinations, accelerating the slow transit may enhance the diagnostic yield by increasing the completion rate or by having a higher proportion of small bowel examined when the examination is incomplete. Even without an increase in the diagnostic yield, the presence of complete small bowel examination is clinically important, as it decreases the likelihood of small bowel pathology.

We found that the use of prucalopride was associated with an increase in the diagnostic yield, 75.8% versus 44%. This increase in the diagnostic yield is, however, higher than what to be expected by the increase in the completion rate in the prucalopride group, although there was a higher yield in complete VCE versus incomplete VCE examinations (65% versus 37%). Our study was not powered or specifically designed to evaluate for a difference in the diagnostic yield as we could not account for all variables that may affect the diagnostic yield such as the quality of bowel preparation, time from the bleeding event to VCE, and variability in reporting VCE findings. A large prospective study would be required to evaluate the effect of significant shortening of the transit time on the diagnostic yield.

Our study has limitations that should be considered, including the retrospective design and the relatively small sample size. The difference in the SBTT between the prucalopride and the control group was, however, substantial. While we found no differences between the two study groups with regard to the baseline characteristics for the collected variables, we could not collect data on the level of physical activity. At least one previous study correlated the level of physical activity with the completion rate [33]. Since all patients were hospitalized with comparable medical comorbidities, it would be unlikely that a big difference in physical activity existed.

In conclusion, this is the first study to evaluate the effect of prucalopride on SBTT in patients undergoing VCE. Our results suggest that the administration of prucalopride at the time of VCE ingestion is a simple and effective intervention to shorten small bowel transit in hospitalized patients. Future studies, preferably randomized controlled trials, are needed to confirm these results.

## Figures and Tables

**Table 1:** Baseline characteristics of study population, the prucalopride versus the control group.

Patient characteristic	Prucalopride *n* = 29	Control *n* = 25	*p* value
Age mean, year (SD)	64.4 (13.9)	65.2 (16.4)	0.85
Male	20 (68.9%)	15 (60%)	0.49
Indication			1
Overt OGIB	26 (89.6%)	23 (92%)	
Occult OGIB	1 (3.4%)	1 (4%)	
Abnormal radiology	2 (6.8%)	1 (4%)	
Medical comorbidities			
Cardiac disease	11 (37.9%)	12 (48%)	0.52
Congestive heart failure	5 (17.2%)	7 (28%)	0.37
Valvular heart disease	8 (27.5%)	5 (20%)	0.46
Cerebrovascular disease	3 (10.3%)	5 (20%)	0.45
Chronic kidney disease	4 (13.7%)	1 (4%)	0.35
Chronic lung disease	2 (6.8%)	2 (8%)	1
Liver cirrhosis	3 (10.3%)	0 (0%)	0.23
Diabetes	8 (27.5%)	7 (28%)	0.97
Antiplatelets (ASA and/or plavix)	11 (37.9%)	7 (28%)	0.44
NSAIDS	2 (6.8%)	2 (8%)	1
Anticoagulants	6 (20.6%)	5 (20%)	0.95
Narcotics	3 (10.3%)	1 (4%)	0.61

SD, standard deviation; OGIB, obscure gastrointestinal bleeding; NSAIDS, nonsteroidal anti-inflammatory drugs.

**Table 2:** Transit times and completion rates in the prucalopride versus the control group.

Outcome	Prucalopride	Control	*p* value
SBTT median, minutes (IQR, range)	92 (63–195, 29–357)	275.5 (263.5–299.5, 152–383)	<0.001
GTT median, minutes (IQR, range)	30 (11–46, 0–116)	27, (13–60, 2–240)	0.61
SB completion rate	93.1%	76%	0.12

SBTT, small bowel transit time; IQR, interquartile range; GTT, gastric transit time; SB, small bowel.

**Table 3:** Summary of positive findings.

	Prucalopride (*n* = 29)	Control (*n* = 25)
All positive findings	22	11
Angiodysplasia	12	4
Ulcer/erosions	4	3
Fresh blood	6	2
Mass	0	2

## Abbreviations

VCE: Video capsule endoscopy
SBTT: Small bowel transit time
GTT: Gastric transit time.

## References

[1] de Leusse A., Vahedi K., Edery J. (2007). Capsule endoscopy or push enteroscopy for first-line exploration of obscure gastrointestinal bleeding?. *Gastroenterology*.

[2] Dionisio P. M., Gurudu S. R., Leighton J. A. (2010). Capsule endoscopy has a significantly higher diagnostic yield in patients with suspected and established small-bowel crohn's disease: a meta-analysis. *American Journal of Gastroenterology*.

[3] Mata A., Llach J., Castells A. (2005). A prospective trial comparing wireless capsule endoscopy and barium contrast series for small-bowel surveillance in hereditary GI polyposis syndromes. *Gastrointestinal Endoscopy*.

[4] Rokkas T., Niv Y. (2012). The role of video capsule endoscopy in the diagnosis of celiac disease: A meta-analysis. *European Journal of Gastroenterology & Hepatology*.

[5] Gerson L. B., Fidler J. L., Cave D. R., Leighton J. A. (2015). ACG clinical guideline: diagnosis and management of small bowel bleeding. *American Journal of Gastroenterology*.

[6] Liao Z., Gao R., Xu C., Li Z. S. (2010). Indications and detection, completion, and retention rates of small-bowel capsule endoscopy: a systematic review. *Gastrointestinal Endoscopy*.

[7] Yazici C., Losurdo J., Brown M. D. (2012). Inpatient capsule endoscopy leads to frequent incomplete small bowel examinations. *World Journal of Gastroenterology*.

[8] Westerhof J., Weersma R. K., Koornstra J. J. (2009). Risk factors for incomplete small-bowel capsule endoscopy. *Gastrointestinal Endoscopy*.

[9] Ben-Soussan E., Savoye G., Antonietti M., Ramirez S., Lerebours E., Ducrotté P. (2005). Factors that affect gastric passage of video capsule. *Gastrointestinal Endoscopy*.

[10] Robinson C. A., Jackson C., Condon D., Gerson L. B. (2011). Impact of inpatient status and gender on small-bowel capsule endoscopy findings. *Gastrointestinal Endoscopy*.

[11] Stanich P., Guido J., Kleinman B., Betkerur K., Porter K., Meyer M. (2016). Video capsule endoscopy completion and total transit times are similar with oral or endoscopic delivery. *Endoscopy International Open*.

[12] Pennazio M., Santucci R., Rondonotti E. (2004). Outcome of patients with obscure gastrointestinal bleeding after capsule endoscopy: report of 100 consecutive cases. *Gastroenterology*.

[13] Carey E. J., Leighton J. A., Heigh R. I. (2007). A single-center experience of 260 consecutive patients undergoing capsule endoscopy for obscure gastrointestinal bleeding. *American Journal of Gastroenterology*.

[14] Apostolopoulos P., Liatsos C., Gralnek I. M. (2007). Evaluation of capsule endoscopy in active, mild-to-moderate, overt, obscure GI bleeding. *Gastrointestinal Endoscopy*.

[15] Singh A., Marshall C., Chaudhuri B. (2013). Timing of video capsule endoscopy relative to overt obscure GI bleeding: Implications from a retrospective study. *Gastrointestinal Endoscopy*.

[16] Koulaouzidis A., Giannakou A., Yung D. E., Dabos K. J., Plevris J. N. (2013). Do prokinetics influence the completion rate in small-bowel capsule endoscopy? A systematic review and meta-analysis. *Current Medical Research and Opinion*.

[17] Kotwal V. S., Attar B. M., Gupta S., Agarwal R. (2014). Should bowel preparation, antifoaming agents, or prokinetics be used before video capsule endoscopy? A systematic review and meta-analysis. *European Journal of Gastroenterology & Hepatology*.

[18] Rokkas T., Papaxoinis K., Triantafyllou K., Pistiolas D., Ladas S. D. (2009). Does purgative preparation influence the diagnostic yield of small bowel video capsule endoscopy?: a meta-analysis. *American Journal of Gastroenterology*.

[19] Briejer M. R., Bosmans J.-P., Van Daele P. (2001). The in vitro pharmacological profile of prucalopride, a novel enterokinetic compound. *European Journal of Pharmacology*.

[20] Lepard K. J., Ren J., Galligan J. J. (2004). Presynaptic modulation of cholinergic and non-cholinergic fast synaptic transmission in the myenteric plexus of guinea pig ileum. *Neurogastroenterology & Motility*.

[21] Kessing B. F., Smout A. J. P. M., Bennink R. J., Kraaijpoel N., Oors J. M., Bredenoord A. J. (2014). Prucalopride decreases esophageal acid exposure and accelerates gastric emptying in healthy subjects. *Neurogastroenterology & Motility*.

[22] Emmanuel A. V., Kamm M. A., Roy A. J., Antonelli K. (1998). Effect of a novel prokinetic drug, R093877, on gastrointestinal transit in healthy volunteers. *Gut*.

[23] Bouras E. P., Camilleri M., Burton D. D., McKinzie S. (1999). Selective stimulation of colonic transit by the benzofuran 5HT4 agonist, prucalopride, in healthy humans. *Gut*.

[24] Bouras E. P., Camilleri M., Burton D. D., Thomforde G., McKinzie S., Zinsmeister A. R. (2001). Prucalopride accelerates gastrointestinal and colonic transit in patients with constipation without a rectal evacuation disorder. *Gastroenterology*.

[25] Saurin J.-C., Delvaux M., Gaudin J.-L. (2003). Diagnostic value of endoscopic capsule in patients with obscure digestive bleeding: blinded comparison with video push-enteroscopy. *Endoscopy*.

[26] Triantafyllou K., Kalantzis C., Papadopoulos A. A. (2007). Video-capsule endoscopy gastric and small bowel transit time and completeness of the examination in patients with diabetes mellitus. *Digestive and Liver Disease*.

[27] Hale M., Drew K., Sidhu R., McAlindon M. (2016). Does magnetically assisted capsule endoscopy improve small bowel capsule endoscopy completion rate? A randomised controlled trial. *Endoscopy International Open*.

[28] Liao Z., Li F., Li Z.-S. (2008). Right lateral position improves complete examination rate of capsule endoscope: A prospective randomized, controlled trial. *Endoscopy*.

[29] Tontini G. E., Wiedbrauck F., Cavallaro F. (2017). Small-bowel capsule endoscopy with panoramic view: results of the first multicenter, observational study (with videos). *Gastrointestinal Endoscopy*.

[30] Flach S., Scarfe G., Dragone J. (2016). A Phase I Study to Investigate the Absorption, Pharmacokinetics, and Excretion of [14C]Prucalopride After a Single Oral Dose in Healthy Volunteers. *Clinical Therapeutics*.

[31] Buscaglia J. M., Kapoor S., Clarke J. O. (2008). Enhanced diagnostic yield with prolonged small bowel transit time during capsule endoscopy. *International Journal of Medical Sciences*.

[32] Westerhof J., Koornstra J. J., Hoedemaker R. A., Sluiter W. J., Kleibeuker J. H., Weersma R. K. (2012). Diagnostic yield of small bowel capsule endoscopy depends on the small bowel transit time. *World Journal of Gastroenterology*.

[33] Shibuya T., Mori H., Takeda T. (2012). The relationship between physical activity level and completion rate of small bowel examination in patients undergoing Capsule endoscopy. *Internal Medicine*.

